# Epidemiology of hyperglycemia during pregnancy in Ethiopia: prevalence, associated factors, and feto-maternal outcomes: systematic review and meta-analysis

**DOI:** 10.1186/s13643-024-02526-z

**Published:** 2024-04-29

**Authors:** Assefa Tola, Nega Assefa, Yadeta Dessie, Lemessa Oljira, Lemma Demissie Regassa, Tadesse Gure, Tesfaye Gobena

**Affiliations:** 1 School of Public Health, College of Health and Medical Sciences, Haramaya University, Harar, Ethiopia; 2 School of Nursing and Midwifery, College of Health and Medical Sciences, Haramaya University, Harar, Ethiopia; 3 School of Medicine, College of Health and Medical Sciences, Haramaya University, Harar, Ethiopia; 4 Department of Environmental Health Science, College of Health and Medical Sciences, Haramaya University, Harar, Ethiopia

**Keywords:** Hyperglycemia in pregnancy, Gestational diabetes mellitus, Burden, Maternal outcomes, Fetal outcomes, Ethiopia

## Abstract

**Background:**

Hyperglycemia in pregnancy (HIP) is a significant medical complication affecting pregnant women globally and is considered a public health burden due to the negative outcomes it can cause for both mother and infant. The aim of this systematic review and meta-analysis was to examine the prevalence, risk factors, and feto-maternal outcomes of HIP in Ethiopia.

**Methods:**

To gather relevant information for this study, both published and unpublished studies were searched for in several major databases, including PubMed, Embase, HINARI, Web of Science direct, and Google Scholar, as well as other sources. The Joanna Briggs Institute (JBI) tool was used to evaluate the methodological quality of the findings from these studies. Data was then extracted and summarized using a template in Microsoft Excel software, and the extracted data was analyzed using Stata software version 16.0. If significant heterogeneity was found between studies, subgroup analyses were conducted to further examine the data.

**Result:**

Eighteen studies were included in this systematic review and meta-analysis, involving a total sample size of 50,816 pregnant women in Ethiopia. The prevalence of HIP among pregnant women varied considerably across the primary studies, ranging from 0.4 to 26.2%. The pooled prevalence of HIP among pregnant women in Ethiopia was found to be 6.9% (95% C 2.2–11.6). Pregnant women with a family history of diabetes had 2.5 times higher odds of developing HIP compared to those without a family history of diabetes (OR = 2.49; 95% CI = 2.02, 2.96). However, there was no significant association found between HIP and maternal obesity (OR 2.31, 95% CI = 0.85, 3.78) or previous history of abortion (OR 3.89; 95% CI 0.85, 6.94). The common fetal outcomes associated with HIP were admission to the intensive care unit (46.2; 95% CI 27.4, 65.1), macrosomia (27.3%; 95% CI 9.4%, 45.1%), and preterm birth (16.9; 95% CI 12.5, 21.3). Additionally, hypertensive disorders of pregnancy (28.0%; 95% CI 15.2, 40.8) and operative delivery (51.4%; 95% CI 35.9, 66.8) were more common among women with HIP in Ethiopia.

**Conclusion:**

Although there was some variation between studies, the meta-analysis revealed that approximately seven out of 100 pregnant women in Ethiopia had HIP. A family history of diabetes was found to be a significant predictor of HIP in Ethiopia. Additionally, HIP was associated with various serious adverse outcomes for both mothers and infants in Ethiopia. These findings highlight the need for national guidelines to ensure that pregnant women are uniformly screened for HIP.

## Background

Hyperglycemia in pregnancy (HIP) is one of the most common medical complications of pregnancy that encompasses various forms of glucose intolerance seen during pregnancy [1]. The World Health Organization (WHO) classifies HIP as diabetes mellitus in pregnancy and gestational diabetes mellitus (GDM) [2, 3]. Diabetes mellitus in pregnancy differs from GDM in that the hyperglycemia is more severe and does not resolve after pregnancy [3]. Whereas in GDM, hyperglycemia is generally mild, first recognized during pregnancy, and does not persist after delivery in most patients [2, 3]. Gestational diabetes mellitus [4] accounts for 90–95% of all cases of diabetes occurring pregnant women and approximately 7% of all pregnancies are complicated by GDM [5, 6].

Diabetes during pregnancy affects an estimated 15% of the pregnant women and low- and middle-income countries bear the highest burden. It is one of the challenging health problems of sub-Saharan African countries with 14% prevalence in the region [7, 8]. In Ethiopia, a prevalence of 13% and 5% were reported among urban and rural women respectively [9].

More than 200,000 cases of GDM occur annually. Gestational diabetes mellitus has increased risk for perinatal morbidity and the risk for type 2 diabetes mellitus. The prevalence may range from 1 to 14% of all pregnancies, depending on the population studied and the diagnostic tests applied [10]. The highest prevalence of GDM is reported from Middle East and North Africa (12.9%) followed by Southeast Asia (11.7%) and Europe had the lowest prevalence (5.8%) with considerable variations observed both within and between countries [11]. Low- and middle-income countries bear the highest burden of GDM with 87.6% of the hyperglycemia in these countries. The prevalence of GDM in Asia is 11.5% [12] and in Africa 13.6% [13]. Current evidence indicated that prevalence of GDM in Sub-Saharan Africa is increasing [14]. In sub-Saharan Africa, its prevalence was high that ranged from 9 to 14%. In Ethiopia, a prevalence of 13% and 5% were reported among urban and rural women respectively [7, 8, 15, 16].

The factors associated with GDM include advanced age, obesity, family history of diabetes, history of GDM, maternal diabetes history, hypertensive history of pregnancy, multipara women and number of abortions, pregestational smoking, pregestational smoking, low physical activity, inadequate dietary diversity, and antenatal depression [4, 13, 16–18],

Since GDM is related to substantial short- and long-term adverse health outcomes, it has become one of the leading causes of mortality and morbidity for both the mother and the infant worldwide. Adverse maternal outcomes including preeclampsia, pregnancy-induced hypertension, recurrent vulvo-vaginal infections, increased incidence of operative deliveries, and obstructed labor were increased among women who suffer from GDM [19]. In addition, the development of diabetes mellitus later in life, the risk of premature rupture of membranes (PROM), antepartum hemorrhage (APH), and postpartum hemorrhage (PPH) are also associated with GDM [20–22]. The negative impact of GDM is encountered not only by women but also a challenge for offspring. Infants born to women with GDM are more likely to have a birth weight ≥ 4.0kgs and this carries 6 times greater risk for birth traumatism and 20 times greater risk for plexus brachialis injuries [23, 24].

National representative epidemiologic data are crucial to understand the burden, major risk factors, and the outcome of GDM in Ethiopia. The recognition of risk factors of GDM is important to identify women at risk, making an early diagnosis, early treatment, and prevention of adverse maternal and perinatal complications. To the best of author’s knowledge, there are no studies that summarize the data on the prevalence and the risk factors of GDM in Ethiopia. This suggests the need for synthesizing those findings already known from previous studies. Therefore, the aim of this systematic review and meta-analysis is to examine the prevalence, risk factors, maternal outcomes, and perinatal outcomes of HIP. The finding will be helpful to develop national and regional policies to address the burden of adverse maternal and perinatal outcomes associated with GDM.

## Methods

### The protocol and registration

The results of this systematic review and meta-analysis were reported using the Preferred Reporting Items for Systematic Review and Meta-Analysis for accuracy statement (PRISMA) guideline, which is a widely recognized standard [25]. We adhered to the flowchart outlined in the PRISMA guideline recommendation to illustrate the selection process from the initial identification of records to the final inclusion of studies. The protocol for this study has been registered on the International Prospective Register of Systematic Reviews (PROSPERO) with the registration number CRD42021289831.

### Inclusion criteria

#### Participants

All studies that reported the occurrence of HIP among pregnant women in Ethiopia considered in this review. Studies that included both pregnant and non-pregnant women were included if data from pregnant women could be separately reported and extracted.

#### Condition

The current review considered studies that reported any of the outcome variables. The outcome variables were prevalence, risk factors, maternal outcomes, and perinatal outcomes of gestational diabetes among mothers and newborns in Ethiopia. Hyperglycemia in pregnancy is defined as “any degree of glucose intolerance that includes DM in pregnancy and GDM” [26]. The main associated factors are advanced maternal age, urban residence, physical inactivity, obesity, dietary diversity, family history of type 2 diabetes mellitus, previous GDM, multigravida, previous fetal macrosomia, pervious unexplained still birth, previous still birth, and polyhydramnios. The primary maternal outcomes were hypertensive disorders of pregnancy, obstructed labor, operative deliveries, antepartum hemorrhage (APH), postpartum hemorrhage (PPH), premature rupture of membranes (PROM), and recurrent infections. The primary fetal outcomes were macrosomia, neonatal trauma, preterm birth, stillbirth, perinatal asphyxia, shoulder dystocia, respiratory distress, and increased admission to neonatal intensive care units.

#### Context

Only studies conducted in Ethiopia were included in the current review. Both community-based and institution-based studies on HIP in Ethiopia were included in this review.

#### Types of studies

All observational studies such as cross-sectional, case–control, and cohort studies conducted in Ethiopia up to October 30, 2021, and written in English language were included in the current review.

### Exclusion criteria

The following studies were not considered: editorials, letters to the editor, commentaries, and case series with less than 30 participants; studies without relevant data to compute the effect sizes of prevalence, determinants, and outcomes of GDM; systematic reviews were not eligible for inclusion; however, their references were screened for relevant primary studies: duplicate studies: here, the most comprehensive and/or recent study with the largest sample size was considered and studies with incomplete data, even after request from the corresponding author.

### Search strategy

All published studies were searched in the following major databases: PubMed, Embase, Health InterNetwork Access to Research Initiative (HINARI), and Web of Science direct (web of science core collection). The search for published studies was not restricted by time, and all published articles up to October 30, 2021, were included in the review. In addition, we performed a manual search to retrieve unpublished studies and grey literature via Google Scholar and other sources including national conference papers and national university repositories. A reverse-forward citation tracking was also done to retrieve additional articles from the reference list of already identified studies. The Boolean operators “AND” and “OR” were used to combined relevant Medical Subject Heading (MeSH) terms.

A compressive search strategy has been employed using the following keywords, alone or in combination, using the Boolean method: “Gestational Diabetes mellitus” OR “gestational diabetes” OR “hyperglycemia in pregnancy” OR “impaired glucose tolerance” OR “Diabetes in pregnancy” OR “pregnancy induced diabetes” OR “gestational hyperglycemia” OR “gestational glucose intolerance” AND “risk factors” OR “determinant factors” OR “associated factors” AND “maternal outcomes” OR “perinatal outcomes” OR “Neonatal outcomes” OR “Birth outcomes” OR “Fetal outcomes” AND “Ethiopia.”

### Study selection

All retrieved articles were exported to the EndNote 20 citation manager and duplicated studies were excluded. In order to guide the study selection process, first tool was developed according to eligibility criteria. Then, the titles and abstracts of papers retrieved from the search were carefully screened, and the full text of potentially eligible articles retrieved. This task was conducted independently by two reviewers (DE and LDR), who further reviewed the full texts of potential articles for final inclusion. The authors compared their results at every step of the selection process, and discrepancies were resolved through discussion and consensus. A third author (ATG) was consulted in case of any disagreement. In the event of unclear or ambiguous information, the corresponding author of the said study was contacted for clarification.

### Data items and extraction

After the selection of the eligible studies, data were extracted and summarized by two investigators independently (LDR and ATG) using a data extraction template in Microsoft excel software. The extracted items were as follows: the last name of the first author, year of the study published, study title, study objective, study setting, study design, sample size, sampling techniques, data collection techniques, response rate, mean or median age and age range in years, screening criteria (universal vs selective), test approach (one step vs two step), screening method (FBS vs OGTT), gestational age during diagnosis, proportion and number of mothers with HIP, risk factors, and adverse maternal outcomes, as well as proportion and number of newborns with perinatal outcomes in the respective studies. To assess the risk factor analyses, we documented the number of HIP cases exposed to a given risk factor (as well as the total number of exposed subjects) and number of cases unexposed to the risk factor (as well as the total number of unexposed subjects). To examine the adverse impacts of HIP, we also noted down the number of cases of each outcome exposed to HIP (as well the total exposed to HIP) and number of cases of the outcome not exposed to HIP (and the total unexposed to HIP). In addition, the measure of association (odds ratio or relative risk with their respective CIs) for each associated factor were extracted and specification made if obtained from a bivariate or multivariate analysis. In case of multivariate analysis, the variables adjusted for were obtained. After the data extraction was completed, the accuracy of the extracted data was verified by comparing the consistency between the extracts. Any dissimilarity and inconsistencies were resolved among the authors by discussion and repeating the procedure. The Preferred Reporting Items for Systematic Reviews and Meta-Analyses (PRISMA) guideline were followed throughout the review and analysis processes [27].

### Assessment of methodological quality and risk of bias

The methodological quality of the included studies was critically evaluated using the quality assessment tool for observational studies (cross-sectional, case–control, and cohort studies) developed by the Joanna Briggs Institute (JBI) [28]. The authors (LDR and ATG) independently evaluated the quality of the studies. Any disagreement was resolved by consensus after they come together and discuss on the issue. The included studies were evaluated against each indicator of the tool and categorized as high (above 80%), moderate (between 60 and 80%), and low quality (low quality below 60%). Studies with a score greater than or equal to 60% were included. Bias was assessed using the Risk of Bias Tool for Prevalence Studies developed by Hoy, Brooks, Woolfe et al., adapted specifically for this systematic review [29]. The tool consists of ten items that assess sampling, attrition, measurement, and reporting bias. The items assess both external and internal validity. Accordingly, items 1–4 assess the external validity of the studies (domains are selection and non-response bias) and items 5–10 assess the internal validity of the studies (items 5–9 assess the domain of measurement bias, and item 10 assesses bias related to analysis). Each study was rated as having a low, moderate, or high risk of bias. When the information provided was not adequate to assist in making judgment for a certain item, we agreed to grade that item with a “NO” meaning high risk of bias. Studies were classified as having a low risk, moderate risk, and high risk of bias when eight or more, six to seven, and five or fewer questions were answered as “yes” respectively. The risk of bias in included studies was presented in a tabular form. Funnel plots and Egger’s test were carried out to check the symmetry that can determine the presence of publication bias [30]. We also employed Egger’s and the Begg’s test to determine if there was significant publication bias. A *p*-value of less than 0.05 was considered significant publication bias.

### Data synthesis and analysis

Stata software version 16.0 was utilized to analyze the extracted data. A meta-analysis was performed on comparable studies with identical variables to calculate the pooled effect sizes namely prevalence for outcomes and odds ratios [31] and their 95% CIs for risk factor from included studies. A random-effect meta-analysis model was utilized to combine study-specific estimates and determine the overall prevalence, risk factors, and adverse outcomes of HIP across multiple studies. This approach yielded a pooled effect size with a 95% confidence interval. The impact of selected independent factors was assessed and presented using a forest plot.

The Cochran *Q*-test and *I*-squared (*I*^2^) statistic were utilized to evaluate the heterogeneity among the studies that were included during this period [32]. The *I*-squared was used to calculate the percentage of total variation in the study estimated due to heterogeneity. The *I*^2^ values of 25%, 50%, and 75% represent low, medium, and substantial heterogeneity, respectively [32, 33]. To investigate the origin of heterogeneity in the studies included in the systematic review, sensitivity analysis, subgroup analyses, and meta-regressions were conducted. Subgroup analyses were performed when there was substantial clinical and/or methodological heterogeneity, utilizing specific variables including screening method, location of the study, year of publication, risk of bias, and study design. A significant difference between subgroups was determined if the *p* value was less than 5%.

## Results

### Selection of the studies

**Fig. 1:**
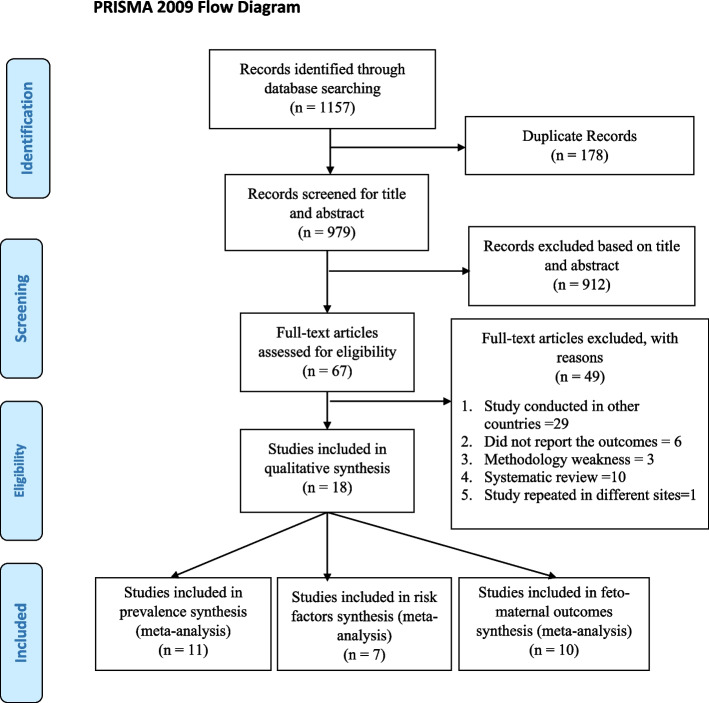
PRISMA flow diagram of selecting and including studies in systematic review and meta-analysis of burden, determinants, and feto-maternal outcomes of HIP in Ethiopia

The search of the scientific databases yielded a total of 1157 articles, out of which 178 duplicate articles were removed. The inclusion criteria were applied to the titles and abstracts of 979 articles, and 912 articles were found to be irrelevant to the topic and were excluded. The full-text papers of 67 articles were retrieved and evaluated for eligibility, resulting in 21 studies that were eligible for methodological quality assessment. Following the exclusion of three studies due to their weak quality score, a total of 18 studies were included in the final systematic review and meta-analysis [34–51] (Fig. 1).

### Baseline characteristics of studies

The selected studies were published between 1999 and 2021. The geographical distribution of the studies showed that six studies were conducted in Northern Ethiopia [40, 41, 43–46], five studies were in southern Ethiopia [37, 42, 49–51], four studies were in central Ethiopia [34, 35, 38, 39, 52], and three studies were in eastern Ethiopia [36, 47, 48].

Cross-sectional design was the study design used in half of the included studies [37–39, 42, 43, 46, 47, 50, 51], followed by prospective cohort [35, 41, 44, 45], case control [36, 40, 48], and retrospective cohort [34, 49]. The sample size ranged from 162 [39] to 19,797 [35] subjects and the total sample sizes included for this review were 50,816. Regarding the sampling techniques, seven studies used systematic random sampling [34, 39, 40, 42–44, 51], while nine studies included all pregnant women or their charts consecutively [35, 36, 38, 45–50].

The main data collection technique was face-to-face interviews [36, 40, 42, 43, 46–48, 50] followed by medical record review [34, 37–39, 49, 51] while the remaining four studies [35, 41, 44, 45] used interview with medical record review. The response rates ranged from 68 [45] to 100% [34, 35, 38, 45, 49, 51]. Five studies [35, 41, 44, 45, 50] did not report the mean age of the participants. The mean age of the patients in the remaining 13 studies ranged from 25.6 (± SD 4.8) to 33.29 (± SD 5.05) years.

The overview of the baseline characteristics of the 18 studies is presented in Table 1 (author, study area, geographic region, publication year, study design, sample size, sampling technique, data collection methods, response rate, and mean age ± SD).

**Table 1:** Summary characteristics of the included studies, 2021

Authors	Study area	Geographical region	Publication year	Study design	Sample size	Sampling technique	Data collection technique	Response rate	Mean age (+ SD) in year	Main outcome
Alemayehu [34]	Addis Ababa	Central Ethiopia	2020	Retrospective cohort study design	2000	Systematic random sampling	Medical record review	100	27.4 (SD ± 4.7)	Prevalence and risk factors of GDM
Aytenew T [35]	Addis Ababa	Central Ethiopia	2019	Institution based prospective cohort	19,797	All consecutive	Interview and medical record review	100	Not reported	Prevalence and outcomes of pregestational DM
Bekele E. [36]	Harar and Dire Dawa	Eastern Ethiopia	2017	Facility based Unmatched case control study	1834	All consecutive	Face to face Interview	98.7%	25.6 (SD ± 4.8)	Prevalence, risk factors and outcome of GDM
Boka Abdisa [37]	Metu, Illubabor Zone	Southern Ethiopia	2019	Facility based cross-sectional study	346	convenient sampling method	Medical record review	95.6%	30.8 (SD ± 4.7)	Adverse birth outcome and associated factors of DM
Eshetu B [38]	Addis Ababa	Central Ethiopia	2019	A facility-based cross-sectional	14,039	All consecutive	Medical record review	100	30.8 (SD ± 4.7)	Prevalence, birth outcome and associated factors of DM
Ewnetu S [39]	Addis Ababa	Central Ethiopia	2016	Institution based cross sectional study	162	Systematic random sampling	Medical record review	80.2%	30 (SD ± 4.4)	Maternal and perinatal outcome of DM
Feleke BE [40]	Amhara regions	Northern Ethiopia	2017	Case control study	2270	Systematic random sampling	Face to face Interview	99.8%	33.3 (SD ± 5.05)	Determinate factors of GDM
Feleke BE[41]	Amhara regions	Northern Ethiopia	2017	Prospective cohort study	3459	Not specified	Interview and medical record review	85.6%	Not reported	Maternal and neonatal outcomes of GDM
Larebo YM [42]	Hadiya Zone	Southern Ethiopia	2021	An institution based cross-sectional study	420	Systematic random sampling	Face to face Interview	89.4%	29.6 (SD ± 7)	Prevalence and risk factors of GDM
Muche AA [43]	Gondar town	Northern Ethiopia	2019	An institution based cross-sectional study	1027	Systematic random sampling	A face-to-face interview	92.5%	27.2 (SD ± 5.24)	Prevalence and risk factors of GDM
Muche AA [44]	Gondar town	Northern Ethiopia	2020	Prospective cohort study	694	Systematic random sampling	Interview and medical record review	68%	Not reported	Effects of GDM on adverse maternal outcomes
Muche AA [45]	Gondar town	Northern Ethiopia	2020	Prospective cohort study	684	All consecutive	Interview and medical record review	100	Not reported	Effects of GDM on adverse neonatal outcomes
Seyoum B. [46]	Eastern zone of Tigray Region	Northern Ethiopia	1999	A community-based survey	890	All consecutive	Interview and measurements	95%	27.5 (SD ± 7.1)	Prevalence of GDM
Wakwoya EB [47]	Harar and Dire Dawa	Eastern Ethiopia	2019	Institution based comparative cross-sectional study	1834	All consecutive	Face to face Interview	98.7%	25.6 (SD ± 4.8)	Adverse pregnancy outcomes and associated factors of GDM
Wakwoya EB [48]	Harar and Dire Dawa	Eastern Ethiopia	2018	Case control study	1834	All consecutive	Face to face Interview	98.7%	25.6 (SD ± 4.8)	Adverse maternal outcomes and associated factors of GDM
Wolka E [49]	Wolaita Zone	Southern Ethiopia	2019	Retrospective cohort study	408	All consecutive	Medical record review	100%	28.9 (SD ± 5.0)	Magnitude of preexisting DM
Wolka E [50]	Wolaita Zone	Southern Ethiopia	2019	Institution based cross sectional study	518	All consecutive	Face to face Interview	91.8%	Not reported	Prevalence and risk factors of GDM
Wolka E [51]	Wolaita Zone	Southern Ethiopia	2019	Institution based cross sectional study	600	Systematic random sampling	Medical record review	100	26.8 (SD + 5.1)	Effects of GDM on pregnancy and birth outcomes

### Reported prevalence of hyperglycemia during pregnancy in Ethiopia

#### Methods of assessing hyperglycemia during pregnancy

Eleven studies reported the prevalence of HIP [34–36, 38, 42–44, 46, 48, 50, 51]. The aims of seven studies were to assess the prevalence of GDM and associated factors among pregnant women [34, 36, 38, 42, 43, 46, 50], while two studies were aimed to assess the prevalence of pre-gestational diabetes [35, 51].

Considerable variation existed among the studies in terms of screening criteria, testing approach, screening methods, and the gestational age of pregnant women during screening. Except one study which selectively screened high-risk pregnant women [36], other studies applied universal screening whereby all pregnant women were screened or had their medical records reviewed. However, study by Aytenew T and his colleagues did not report the screening strategy they used [35]. Seven studies used a 2-h 75 g OGT test in order to screen pregnant women for GDM [36, 42–44, 46, 48, 50]. Only two studies applied screening for pregnant women during 24–28 weeks of gestation [42, 50] whereas three studies applied screening on all women above 24 weeks of gestations [44, 46, 48].

The methods of screening HIP in these 11 studies are presented in Table 2 (research objective, screening criteria, testing approach, screening methods, gestational age of pregnant women during screening).

**Table 2:** Screening methods for HIP used in the included studies, 2021

Authors	The objective of the study	Screening criteria (recruitment)	Test approach	Screening method	Gestational age during screening
Alemayehu [34]	To determine the magnitude and factors associated with GDM	Document review of all pregnant women	One step	Medical record review	All
Aytenew T [35]	To assess the prevalence of pregestational DM and its pregnancy outcomes	Not reported	Not reported	Medical record review	All
Bekele E.[36]	To assess the prevalence of GDM and its association with maternal and perinatal adverse outcomes among pregnant mothers who gave birth in Hiwot Fana and Dilchora Specialized Referral Hospitals	Selective	One step	2 h 75 g OGTT	All
Eshetu B [38]	To assess the prevalence of DM, birth outcomes, and associated factors among mothers that delivered in Tikur Anbessa Specialized Hospital	Document review of all pregnant women	Not applicable	Medical record review	28 weeks +
Larebo YM [42]	To assess the prevalence of GDM and associated factors among women attending antenatal care in Hadiya Zone public Hospitals, Southern Ethiopia	Universal	One step	2 h 75 g OGTT	24–28 weeks
Muche AA [43]	To determine the prevalence of GDM and associated factors among women attending antenatal care at Gondar town public health facilities, Northwest Ethiopia	Universal	One step	2 h 75 g OGTT	20–23 +
Muche AA [44]	To assess the effects of GDM on the risk of adverse maternal outcomes in Northwest Ethiopia	Universal	One step	2 h 75 g OGTT	24 + weeks
Seyoum B.[46]	To assess the prevalence of GDM in rural pregnant mothers in northern Ethiopia	Universal	One step	2 h 75 g OGTT	24 + weeks
Wakwoya EB [48]	To assess the adverse maternal outcome and its association with GDM among mothers who gave birth at selected public hospitals in Eastern Ethiopia	Universal	One step	2 h 75 g OGTT	24 + weeks
Wolka E [51]	To assess the magnitude of pre-existing DM among pregnant women and identify associated risk factors	Document review of all pregnant women	One step	Medical record review	All
Wolka E [50]	To determine the prevalence of GDM and to identify associated factors in Wolaita Zone, Southern Ethiopia	Universal	One step	2 h 75 g OGTT	24–28 weeks

Studies were categorized according to their risk of bias; three studies (27.3%) had low, 4 (36.4%) had moderate, and 4 (36.4%) had high risk of bias. The studies with high risk of bias had either data collected from hospital records rather than from subjects or unclear measurement protocol (Table 3).

**Table 3:** Risk of bias assessment of included studies, 2021

**Criteria**	Alemayehu GS (2020)	Aytenew T (2019)	Bekele E. (2017)	Eshetu B (2019)	Larebo YM (2021)	Muche AA (2019)	Muche AA (2020)	Seyoum B.(1999)	Wakwoya EB (2018)	Wolka E (2019)	Wolka E (2019)
**External validity**											
1. Was the study target population a close representation of the national pregnant population in relation to relevant variables?	Yes	Yes	Yes	Yes	Yes	Yes	Yes	Yes	Yes	Yes	Yes
2. Was the sampling frame a true or close representation of the target population?	Yes	Yes	Yes	No	Yes	Yes	Yes	Yes	No	No	Yes
3. Was some form of random selection used to select the sample, OR, was a census undertaken?	Yes	Yes	Yes	Yes	Yes	Yes	Yes	Yes	Yes	Yes	No
4. Was the likelihood of non-participation bias minimal?	No	Yes	Yes	Yes	No	No	No	No	No	Yes	No
**Internal validity**											
5. Were data collected directly from the subjects? (as opposed to medical records)	No	No	Yes	No	Yes	Yes	Yes	Yes	Yes	No	Yes
6. Were acceptable diagnostic criteria for GDM used?	No	No	No	No	Yes	Yes	Yes	Yes	Yes	No	Yes
7. Was a reliable and accepted method of testing for GDM utilised?	No	No	Yes	No	Yes	Yes	Yes	Yes	Yes	No	Yes
8. Was the same mode of data collection used for all subjects?	Yes	No	Yes	Yes	Yes	Yes	Yes	Yes	Yes	Yes	Yes
9. Was GDM tested for within the advised gestational period of 24–28 weeks?	No	No	No	No	Yes	No	No	No	No	No	Yes
10. Were the numerator(s) and denominator(s) for the calculation of the prevalence of GDM appropriate?	Yes	Yes	No	Yes	Yes	Yes	Yes	Yes	Yes	Yes	Yes
11. Summary item on the overall risk of study bias	High	High	Moderate	High	Low	Low	Low	Low	Moderate	High	Low

#### The pooled prevalence HIP

**Fig. 2:**
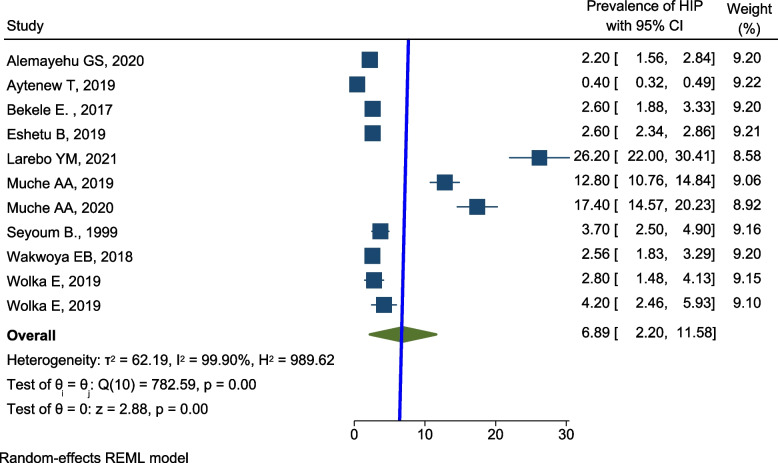
Forest plot for the pooled prevalence of HIP in Ethiopia, 2021

A total of 41,653 pregnant women were included in this meta-analysis. The prevalence of HIP among pregnant women was varied considerably and ranged from 0.4 [35] to 26.2% [42] across reports of primary studies in Ethiopia. The overall pooled prevalence of HIP among pregnant women in Ethiopia was 6.9% (95% CI 2.2–11.6; *I*^2^ = 99.90%) (Fig. 2).

#### Sub-group analysis of HIP

The prevalence of HIP in Ethiopia exhibited significant heterogeneity (*I*^2^ = 99.90%) and *Q* test (Tau-squared = 62.19, *p* < 0.001), which could be attributed to differences in screening methods, study location, year of publication, risk of bias, and study design. Therefore, a subgroup analysis was conducted to evaluate the variability in the prevalence of HIP concerning screening method, study location, and risk of bias. The subgroup analysis indicated significant variability between primary studies regarding the magnitude of HIP based on the geographical location of the study. The study showed that the prevalence of HIP was highest (18.6%; 95% CI 11.0, 26.2; *I*^2^ = 95.17) in western Ethiopia and lowest in central Ethiopia (1.7%; 95% CI 0.3, 3.1; *I*^2^ = 98.80). The pooled prevalence of HIP was higher among studies that utilized a 2-h 75 g OGTT (9.8%; 95% CI 3.1, 16.5; *I*^2^ = 99.49) and lower in studies that used medical record review (1.9%; 95% CI 0.8, 3.1; *I*^2^ = 98.0). The prevalence of HIP was highest (18.6%; 95% CI 11.0, 26.2; *I*^2^ = 95.17) in articles with low-risk bias, followed by studies with moderate-risk bias (2.9%; 95% CI 2.3, 3.6; *I*^2^ = 42.76), and lowest in studies with high-risk bias (1.9%; 95% CI 0.8, 3.1; *I*^2^ = 98.0). Please refer to Table 4 for more information.

**Table 4:** Subgroup analysis of the prevalence of HIP in Ethiopia, 2021

Subgroup	Number of studies	Sample size	Prevalence (95% CI)	Heterogeneity			
				**Q-value**	**Df**	***I***^**2**^	***p*-value**
Geographical distribution							
Central	3	35836	1.7 (0.4, 3.1)	270.7	2	98.8	0.012
Eastern	2	3668	2.6 (2.1, 3.1)	0.01	1	0.00	0.000
Western	3	2141	18.6 (11.0, 26.2)	33.0	2	95.17	0.000
Northern	1	890	3.7 (2.5, 4.9)	0.00	0	-	0.000
Southern	2	1118	3.4 (2.0, 4.7)	1.58	1	36.7	0.000
Screening method							
2 h OGTT	7	7217	9.8 (3.1, 16.5)	294.57	6	99.49	0.004
Medical record	4	36436	1.9 (0.8, 3.1)	280.9	3	98.0	0.001
Risk of bias							
High	4	36,436	1.9 (0.8, 3.1)	280.9	3	98.0	0.001
Moderate	4	5076	3.0 (2.3, 3.6)	5.32	3	42.76	0.000
Low	3	2141	18.6 (11.0, 26.2)	33.00	2	95.17	0.000
Study design							
Case control	2	3668	2.6 (2.1, 3.1)	0.01	1	0.00	0.000
Cohort study	3	22491	6.6 (− 3.9, 17.1)	168.14	2	99.89	0.218
Cross-sectional	6	17494	8.6 (1.2, 15.9)	217.41	5	99.55	0.022
Gestational age specified							
No	4	24231	1.9 (0.8, 3.1)	75.42	3	93.58	0.001
Yes	7	19422	9.8 (3.1, 16.5)	280.9	6	99.67	0.004

**Fig. 3:**
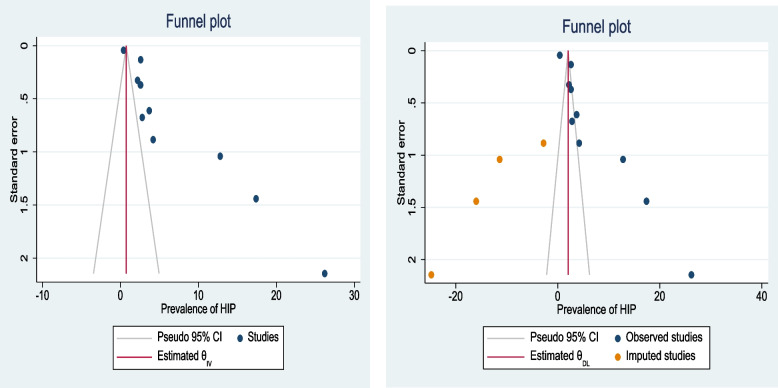
Funnel plots of the prevalence of hyperglycemia during pregnancy in Ethiopia, 2021

The meta-analysis of primary studies included in this study exhibited publication bias (Egger’s test, βo = 12.09, *p*-value < 0.001). The trim and fill analysis addressed this by adding five studies, resulting in a pooled prevalence of HIP in Ethiopia of 2.0% (95% CI 1.9, 2.2) (Fig. 3).

### Factors associated with HIP

A total of seven studies assessed the factors associated factors with HIP in Ethiopia [34, 36, 40, 42, 43, 50, 51] (Table 5). These associated factors can be classified as socio-demographic characteristics, medical and behavioral factors, and past and current obstetric history.

**Table 5:** Factors associated with HIP among mothers in Ethiopia, 2021

Author name	Geographical region	Year of published	Study design	Sample size	Risk factors
Alemayehu [34]	Addis Ababa	2020	Retrospective cohort study	2000	Maternal age, family history of DM, previous CS,
Bekele E. [36]	Harar and Dire Dawa	2017	Unmatched case–control study	1834	Obesity, family history of DM
Feleke BE [40]	Amhara regions	2017	Case–control study	2270	Illiterate, obesity, dietary diversity, family history of DM, previous history of GDM, physical activity, Abortion history, previous CS, history of stillbirth, history of macrosomic baby, history of IUFD, parity
Larebo Y [42]	Hadiya Zone	2021	An institution-based cross-sectional study	420	Urban residence, primary education, coffee drinking, dietary diversity, Abortion history, late GA
Muche AA [46]	Gondar town	2019	An institution-based cross-sectional study	1027	Obesity, dietary diversity, family history of DM, previous history of GDM, physical activity, ANC depression
Wolka E [53]	Wolaita Zone	2019	Institution-based cross-sectional study	600	Abortion history, history of macrosomic baby
Wolka E [54]	Wolaita Zone	2019	Institution-based cross-sectional study	518	Family history of DM, Abortion History, previous CS

#### Socio-demographic characteristics

The main socio-demographic factors which reported by three or more studies and included in this analysis were maternal age, residence, marital status, education status, employment status, and income. Three studies assessed the association between maternal age and HIP [34, 36, 43]. Except study at Addis Ababa [34] which reported unadjusted association, two studies revealed that there was no association between maternal age and HIP. Similarly, three studies assessed the effect of residence on HIP [40, 42, 50]. An institutional-based cross-sectional study in Hadiya zone reported that pregnant women living in urban area were 2 times more likely (AOR 2.18; 95% CI 1.27, 3.73) to develop HIP than rural women [42]. In contrast, the remaining studies did not identify an association between place of residence and HIP. Furthermore, three primary studies examined the association between the educational status of pregnant women and HIP [40, 42, 43]. Two of these studies found that pregnant women with higher educational level had lower chance of developing HIP [40, 42]. Conversely, the other study did not establish a correlation between educational status and HIP. Additionally, none of the primary studies reported any association between HIP and the marital status, employment status, or income of pregnant women.

#### Medical and behavioral factors

The medical factors included in this analysis were coffee drinking, dietary diversity, maternal obesity, chronic hypertension, family history of DM, history of previous GDM, and physical exercise. Only one study reported the positive association between coffee drinking and HIP [42]. The association of HIP with history of previous GDM and physical exercise was reported by two of the primary studies [40, 43]. Three studies reported that dietary diversity had a significant association with the risk of developing HIP in Ethiopia [40, 42, 43].

**Fig. 4:**
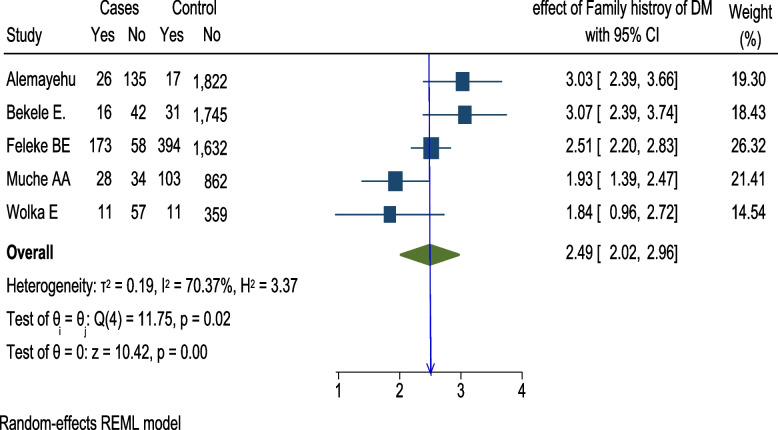
Meta-analysis of the association between hyperglycemia during pregnancy and family history of DM among pregnant women in Ethiopia, 2021

Moreover, a total of 4 articles [36, 40, 43, 50] were included to determine the association of obesity and HIP, and three of the studies [36, 40, 43] had a significant association with HIP. The pooled analysis showed that there was no significant association between maternal obesity and HIP (OR = 2.31; 95% CI = 0.85, 3.78, *I*^2^ = 74.81%). A total of five articles [34, 36, 40, 43, 50] were indicated that family history of DM significantly associated with HIP. The pooled analysis with the random-effect model showed that women with family history of DM had 2.5 times higher odds of developing HIP than women without family history of DM (OR = 2.49; 95% CI = 2.02, 2.96: *I*^2^ = 70.37%, *p*-value < 0.0193) (Fig. 4).

#### Obstetric factors

**Fig. 5:**
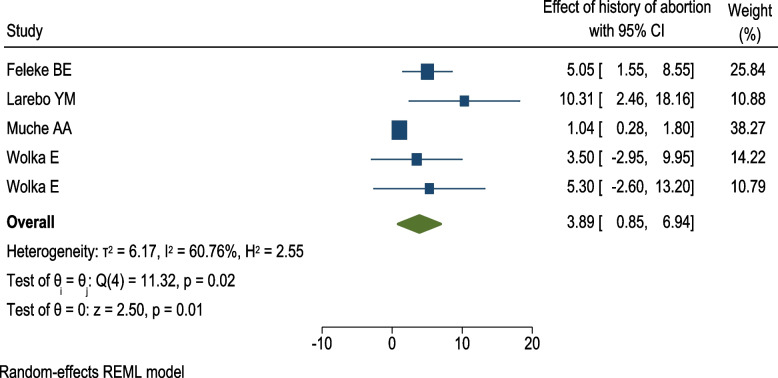
Meta-analysis of the association between hyperglycemia during pregnancy and previous history of abortion among pregnant women in Ethiopia, 2021

The obstetric history included in this analysis were having history of abortion, previous CS, stillbirth, macrosomic baby, and IUFD, current gravida, parity, gestational age, and ANC depression. Three of the included studies reported the significant association between having previous cesarean section and HIP [34, 40, 50]. Having a previous macrosomic baby was one of the factors identified by four studies [40, 43, 50, 51]. Two of these studies [40, 50] reported significant association whereas the other two [43, 51] stated no association between previous history of macrosomic baby and current HIP. Among four studies [34, 36, 40, 43] that assessed the effect of parity on the risk of HIP, only one study [40] reported a significant association. Similarity, out of three studies [40, 43, 50] determined the relationship between having history stillbirth and hyperglycemia during current pregnancy, only one study [40] indicated a significant association. Having a history of abortion was one of the main risk factors for developing HIP that was reported by five studies [40, 42, 43, 50, 51]. Four of these studies indicated that having history of abortion had an association with HIP [40, 42, 50, 51]. However, the pooled analysis showed that there was no association between previous history of abortion and HIP among pregnant women in Ethiopia (OR = 3.89; 95% CI = 0.85, 6.94; *I*^2^ = 60.76%, *p*-value < 0.0122) (Fig. 5).

### Feto-maternal outcomes of hyperglycemia during pregnancy

#### Fetal outcomes of hyperglycemia during pregnancy

The main fetal outcomes reported by the primary studies were macrosomia, preterm birth, stillbirths, admission to ICU, low birth weight, respiratory distress, congenital anomaly, neonatal trauma, perinatal asphyxia, spontaneous abortion, and intrauterine fetal death (Table 6).

**Table 6:** Main fetal outcomes of pregnant women with hyperglycemia during pregnancy in Ethiopia, 2021

Author Name	Location	Year of published	Study design	Sample size	Macrosomia	Preterm birth	Stillbirth	Admission to ICU	Low birthweight	Respiratory distress	Congenital anomalies	Neonatal trauma	Perinatal asphyxia	Abortion	IUFD
Aytenew T [35]	Central Ethiopia	2019	Cohort study	80	9.2%	30.3%	3.8%	53.8%	17.1%	10.9%	3.8%	1.4%		5%	
Boka Abdisa [37]	South West Ethiopia	2019	Cross-sectional study	157	21%	12.1%	0.6%	63.1%	5.7%	10.2%	1.9%	1.3%	8.3%		0.6%
Eshetu B [38]	Central Ethiopia	2019	Cross-sectional study	346	17.6%	17.9%	2.6%	65.3%	10.1%	9.2%		1.7%	10.1%		
Ewnetu S [39]	Central Ethiopia	2016	Cross-sectional study	161	78.4%	18.6%	16%								
Muche AA [44]	Northwest Ethiopia	2020	Cohort study	118	21.2%	22%		33.1%	33.1%						
Wakwoya EB [47]	Eastern Ethiopia	2019	Cross-sectional study	47	31.9%	10.6%	8.5%	14.9%		17%	6.4%				
Wolka E [49]	Southern Ethiopia	2019	Cohort study	136	11.8%	11%	4.4%							0.8%	

All the seven studies [35, 37–39, 44, 48, 49] assessed the magnitude of macrosomia among infants born from pregnant women with HIP. The prevalence ranged from 9.2% which was reported by a study in Addis Ababa [35] to 78.4% reported by another study in Addis Ababa [39].

**Fig. 6:**
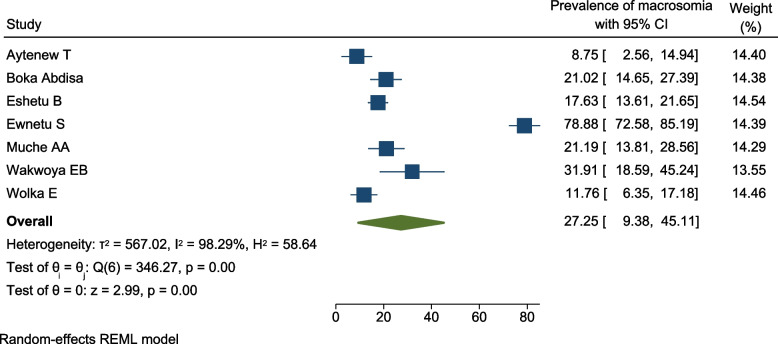
Forest plot for the pooled prevalence of macrosomia among pregnant women with HIP in Ethiopia, 2021

The overall prevalence of macrosomia among newborns from pregnant women with HIP was 27.3% (95% CI 9.4%, 45.1%; *I*^2^ = 98.29%, *p* < 0.01). The percentage of *I*^2^ statistic indicates significant heterogeneity across the studies (*I*^2^ = 98.29%, *p* < 0.01) (Fig. 6).

### Subgroup analysis by study design

Subgroup analysis by study design was performed to minimize heterogeneity. However, the *p*-value of Egger’s regression test indicated the absence of small-study effect at *p* = 0.34.

**Fig. 7:**
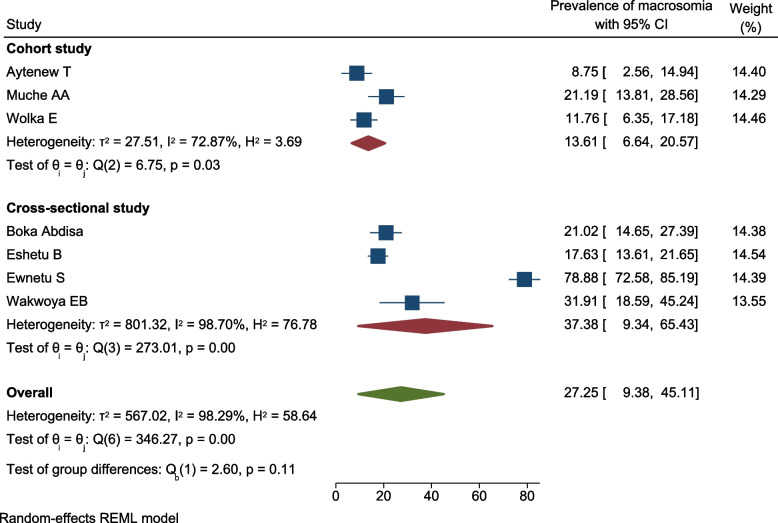
Subgroup analysis showing the prevalence of macrosomia among newborns from pregnant women with hyperglycemia based on the study design in Ethiopia, 2021

According to subgroup analysis study design, the pooled prevalence of macrosomia newborns from among pregnant women with hyperglycemia was higher in cross-sectional studies (37.4%; 95% CI 9.4, 65.5; *I*^2^ = 98.7%, *p* < 0.01) than cohort studies (13.6%; 95% CI 6.6, 20.6; *I*^2^ = 74.87%, *p* < 0.05) (Fig. 7).

**Fig. 8:**
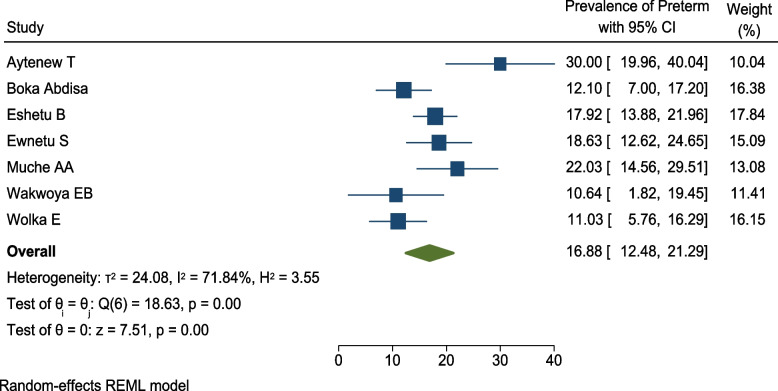
Forest plot for the pooled prevalence of preterm birth among pregnant women with HIP in Ethiopia, 2021

Similarly, the prevalence of preterm birth among pregnant women with HIP in Ethiopia was also reported by seven studies [35, 37–39, 44, 48, 49]. The highest prevalence of preterm birth was 30.2% which reported from study in central Ethiopia [35] and the lowest prevalence was 10.6% which reported by study in Eastern Ethiopia [49]. The pooled prevalence of preterm birth among pregnant mothers with hyperglycemia was 16.9 (95% CI 12.5, 21.3; *I*^2^ = 71.84%, *p* < 0.001). This shows that the included studies had moderate heterogeneity (Fig. 8).

**Fig. 9:**
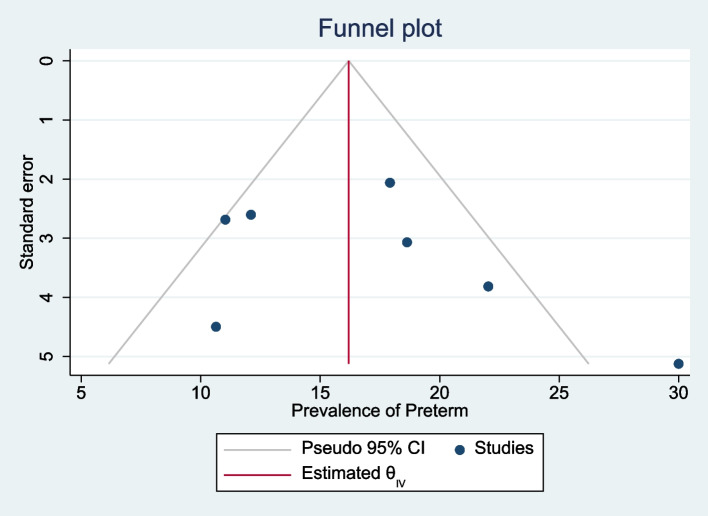
Funnel plots of the prevalence of preterm birth among pregnant women with HIP in Ethiopia, 2021

The symmetry of the funnel plot suggests that there was no publication bias, as well as Egger’s test with a *p*-value of 0.2053 shows the absence of small-study effects (Fig. 9).

**Fig. 10:**
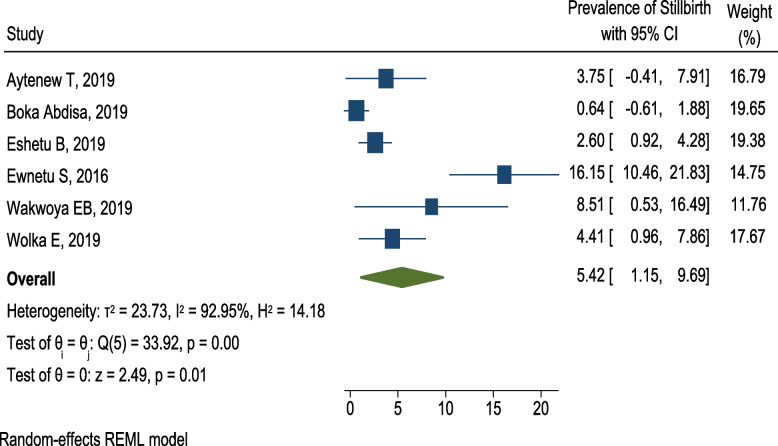
Forest plot for the pooled prevalence of stillbirth among pregnant women with HIP in Ethiopia, 2021

Six articles [35, 37–39, 48, 49] reported the prevalence of stillbirth among pregnant women with hyperglycemia in Ethiopia. The lowest prevalence of stillbirth among pregnant women with hyperglycemia was observed in southwest Ethiopia (0.6%) [37] whereas the highest prevalence of stillbirth was reported in central Ethiopia, Addis Ababa (16.1%) [39]. However, the pooled prevalence of stillbirth among pregnant women with HIP was 5.4 (95% CI 1.2, 9.7; *I*^2^ = 92.95%, *p* = 0.01). This shows that the included studies had considerable heterogeneity (*I*^2^ = 92.95%, *p* = 0.01) (Fig. 10).

**Fig. 11:**
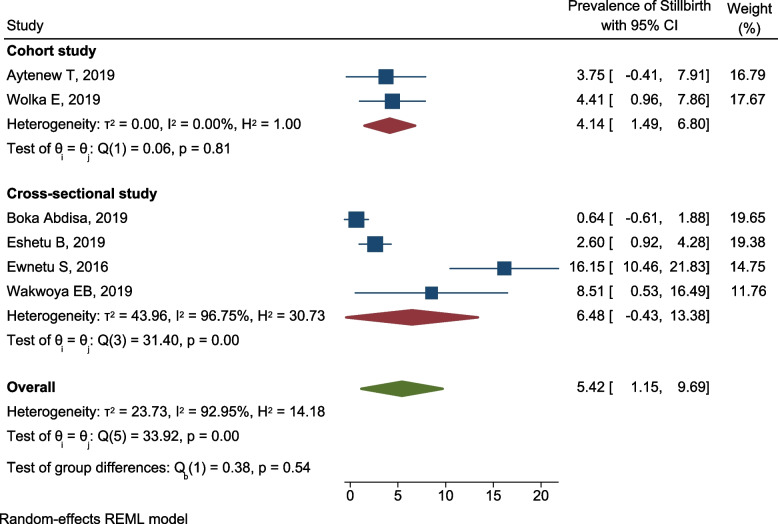
Subgroup analysis showing the prevalence of stillbirth among pregnant women with hyperglycemia based on the study design in Ethiopia, 2021

Subgroup analysis was done to assess variability in the prevalence of stillbirth among women with HIP in relation to study design. This sub-group analysis showed that the prevalence of stillbirth was lower and less heterogenous in cohort studies, 4.1% (95% CI 1.5, 6.8; *I*^2^ = 0.00%), and the highest and more heterogenous in cross-sectional studies, 6.68% (95% CI 0.43, 13.38; *I*^2^ = 96.75) (Fig. 11).

**Fig. 12:**
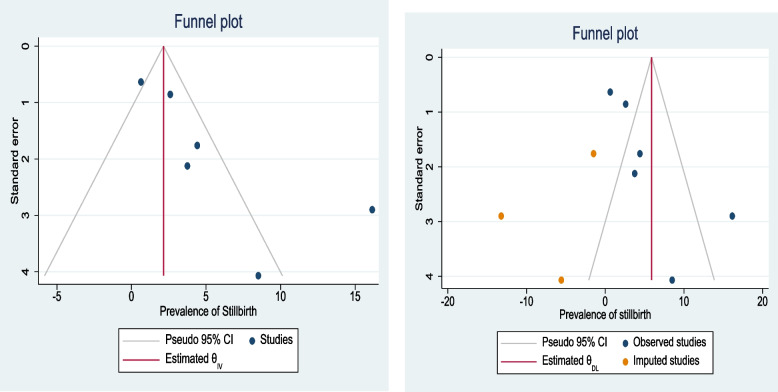
Funnel plots of the prevalence of stillbirth among women with HIP in Ethiopia, 2021

Asymmetrical distribution of the funnel plot implies the presence of publication bias among the included studies (Fig. 12). Furthermore, Egger’s test with a *p*-value of 0.019 shows the presence of publication bias. We have performed trim and fill method analysis. A bias-adjusted effect estimate of stillbirth among pregnant mothers with HIP was found to be 5.4 (95% CI 1.2, 9.6) % in both right and left imputing, assuming there are missing studies (Table 7).

**Table 7:** The prevalence of common fetal outcomes of pregnant women with HIP in Ethiopia, 2021

Fetal outcomes	Number of studies	Sample size	Prevalence (95%CI)	Heterogeneity		Egger’s test
				***I***^**2**^	***p*-value**	
Macrosomia	7	1045	27.3 (9.4%, 45.1)	98.29%	0.01	0.3400
Preterm birth	7	1045	16.9 (12.5, 21.3)	71.84%	0.001	0.2053
Stillbirth	6	927	5.4 (1.2, 9.7)	92.95%	0.01	0.0190
Admission to ICU	5	748	46.2 (27.4, 65.1)	96.47%	0.001	0.2072
Low birthweight	4	701	16.1 (4.6, 27.7)	95.3%	0.0261	0.0261
Respiratory distress	4	630	10.1 (7.7, 12.4)	0.00	0.000	0.1789

Five studies reported the magnitude of ICU admission among neonates from mothers with HIP in Ethiopia. The ICU admission was ranged from 14.9% (95% CI 4.7, 25.1) in eastern Ethiopia to 65.3% (95% CI 60.3, 70.3) in central Ethiopia. The pooled prevalence of ICU admission of neonates from mothers with HIP in Ethiopia was 46.2% (95% CI 2.4, 65.1; *I*^2^ = 96.46%, *p* < 0.01). Due to higher heterogeneity of the studies, subgroup analysis was employed using study design. Hence, subgroup analysis showed that the magnitude of ICU admission among neonates from mothers with HIP in Ethiopia was slightly higher in cross-sectional studies (47.9%, 95% CI 16.0, 80.0; *I*^2^ = 88.37%, *p* = 0.010) than in cohort studies (43.1%, 95% CI 22.8, 63.4; *I*^2^ = 88.37%, *p* = 0.010). The symmetry of the funnel plot indicated that there was no publication bias, as well as Egger’s test with a *p*-value of 0.2072 shows the absence of small-study effects.

Prevalence of low birth among neonates from mothers with HIP in Ethiopia was reported by 4 studies. Accordingly, the lowest prevalence of low birth weight was reported by study in Mettu Karl Hospital Western Ethiopia (5.7% 95% CI 2.1, 9.4) and the highest prevalence was reported by prospective cohort study in western Ethiopia (33.1% 95% CI 24.6, 41.5) with the pooled prevalence of 16.1% (95% CI 4.6, 27.7; *I*^2^ = 95.35%, *p* < 0.01). Similarly, the subgroup analysis indicated that prevalence of low birth was higher in cohort studies (25.3%, 95% CI 10.0, 40.0; *I*^2^ = 84.78%, *p* = 0.01) than cross-sectional studies (8.0, 95% CI 3.7, 12.3; *I*^2^ = 68.40%, *p* = 0.08). The funnel plot was asymmetric with the Egger’s test of 0.0261, which indicated that there was publication and small study effects. Hence, the trim and fill analysis was done, two studies were added, and the total number of the studies becomes 6. The pooled prevalence of low birth weight was 8.1% (95% CI − 1.1, 17.3). On the other hand, four of the studies reported the prevalence of respiratory distress among newborns from mothers with HIP in Ethiopia. The pooled prevalence of respiratory distress among newborns from mothers with HIP in Ethiopia was 10.1% (95% CI 7.7, 12.4; *I*^2^ = 0.00, *p* = 0.00). The funnel plot was symmetric and the Egger’s test was 0.1789 which indicated there was no publication bias and small study effects (Table 7).

### Maternal outcomes of hyperglycemia during pregnancy

Hypertensive disorders of pregnancy (HDP), operative delivery, PROM, APH, PPH, obstructed labor, traumatized labor, polyhydramnios, and hypothyroidism were the main maternal outcomes reported by the eight of the primary studies [35–39, 41, 45, 48, 49]. A total of 1095 pregnant women with HIP were included to determine the magnitude of adverse maternal outcome. A cross-sectional study in Addis Ababa reported the highest number, eight, of adverse maternal outcomes [38]. The highest prevalent adverse maternal outcome among pregnant women with hyperglycemia was HDP, 72.3%, which was reported by a case control study in eastern Ethiopia [49].

Only three studies reported the prevalence of PROM among pregnant women with hyperglycemia in Ethiopia [36, 45, 48]. Based on these studies, the prevalence of PROM among pregnant women with hyperglycemia was 16.5% in north west [45], 40.4% [36], and 59.6% in Eastern Ethiopia [48]. Most of the adverse maternal outcomes were reported only by one or two studies. Accordingly, obstructed labor [38, 49], APH [45, 49], PPH [45, 49], traumatized labor [37, 38], polyhydramnios [37, 38], hypothyroidism [37, 38], and admission to ICU [38] were reported by few studies (Table 8).

**Table 8:** Main maternal outcomes of pregnant women with hyperglycemia during pregnancy in Ethiopia, 2021

Author name	Location	Year of published	Study design	Sample size	HDP	Operative delivery	PROM	Obstructed labor	APH	PPH	Traumatize labor	Polyhydramnios	Hypothyroidism	ICU admission
Aytenew T [35]	Central Ethiopia	2019	Cohort study	80	28.8	61.8%								
Bekele E. [36]	Eastern Ethiopia	2017	Case control study	47	27.7%		40.4%							
Boka A [37]	Western Ethiopia	2019	Cross-sectional study	157	29.9%	67.5%					1.3%	1.9%	2.6%	
Eshetu B [38]	Central Ethiopia	2019	Cross-sectional study	346	26%	57.8%		0.2%			2%	1.4%	1.7%	32.1%
Ewnetu S [39]	Central Ethiopia	2016	Cross-sectional study	161	21.6%	66.4%								
Muche AA [45]	Western Ethiopia	2020	Cohort study	121	12.4%	34.7%	16.5%		16.5%	13.2%				
Wakwoya EB [49]	Eastern Ethiopia	2018	Case control study	47	72.3%		59.6%							
Wolka E [49]	Southern Ethiopia	2019	Cohort study	136	9.6%	20.6%		5.9%	4.4%	0.7%				

**Fig. 13:**
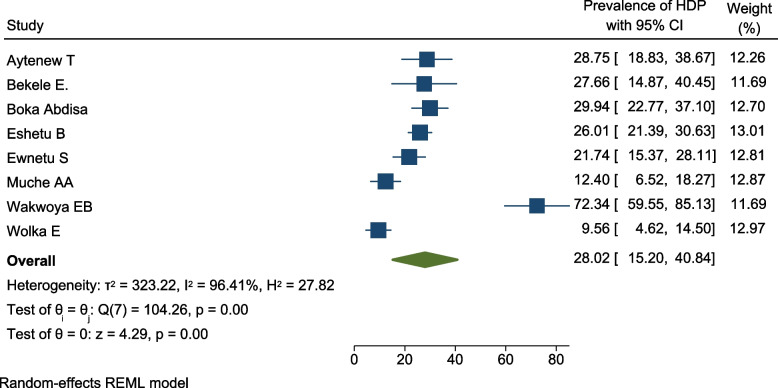
Forest plot for the pooled prevalence of HDP among pregnant women with hyperglycemia during pregnancy in Ethiopia, 2021

All the included studies reported the burden of HDP among pregnant women with hyperglycemia [35–39, 41, 45, 48, 49]. The magnitude varied from 9.6 [49] to 72.3% [48]. However, most of the included studies stated the prevalence between 21 and 29% [35–39]. The pooled prevalence of HDP among pregnant women with hyperglycemia in Ethiopia was 28.0% (95% CI 15.2, 40.8; *I*^2^ = 96.41%, *p* < 0.001). This shows that the included studies had considerable heterogeneity (Fig. 13). Hence, subgroup analysis by study design was undertaken in order to minimize heterogeneity between studies.

**Fig. 14:**
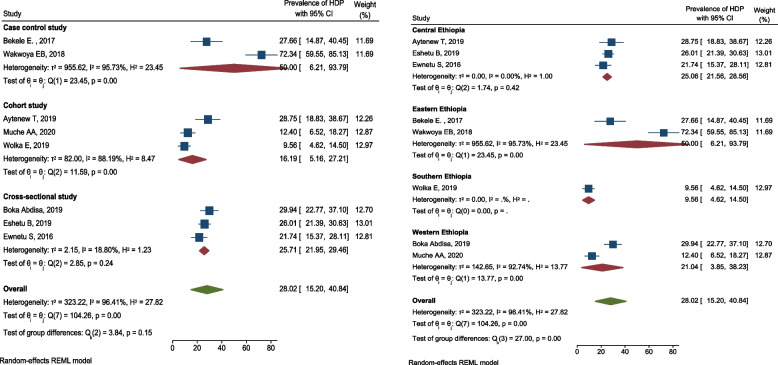
Subgroup analysis showing the prevalence of HDP among pregnant women with hyperglycemia based on the study design and the study location in Ethiopia, 2021

The pooled prevalence of HDP among pregnant women with HIP was higher in case control studies (50%; 95% CI 6.2, 93.8; *I*^2^ = 95.73), and lower in cohort studies (16.2%; 95% CI 5.2, 27.2; *I*^2^ = 88.19) (Fig. 14).

**Fig. 15:**
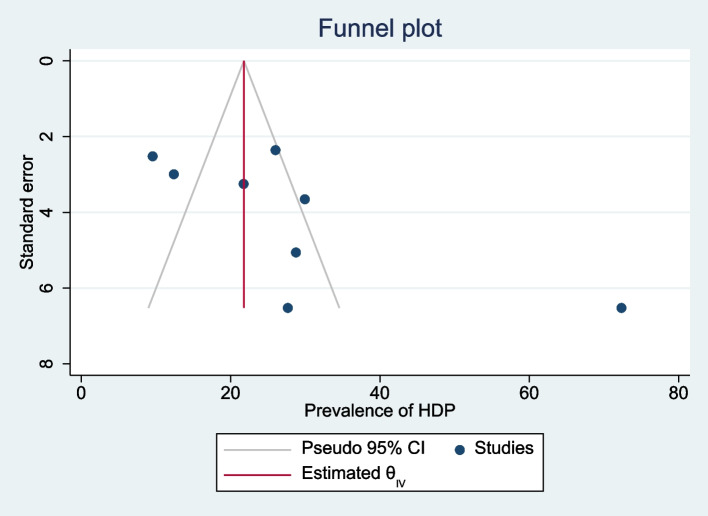
Funnel plots of the prevalence of HDP among pregnant women with HIP in Ethiopia, 2021

There was a publication bias (Egger’s test, βo = 7.87, *p*-value = 0.011) among primary studies included in this meta-analysis. The trim and fill analysis added three studies and the pooled prevalence of HDP among pregnant women with HIP in Ethiopia become 18.7% (95% CI 8.8, 28.5) (Fig. 15).

**Fig. 16:**
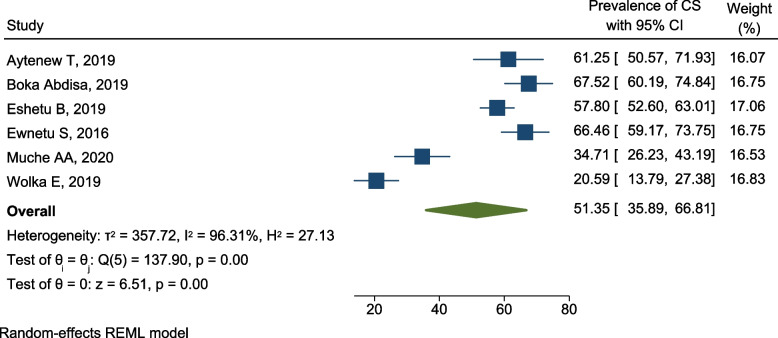
Forest plot for the pooled prevalence of cesarean section among pregnant women with HIP in Ethiopia, 2021

Similarly, six studies reported the prevalence of operative delivery (cesarean section) among pregnant women with hyperglycemia in Ethiopia [35, 37–39, 45, 49]. According to these studies, at least one out five pregnant women with hyperglycemia would undergone cesarean section. The proportion of pregnant women with hyperglycemia who underwent cesarean section was highest in study conducted in south west Ethiopia, 67.5% [37], and lowest in southern Ethiopia, 20.6% [49]. The pooled prevalence of cesarean section among pregnant women with hyperglycemia in Ethiopia was 51.4% (95% CI 35.9, 66.8; *I*^2^ = 96.11%, *p* < 0.001) (Fig. 16).

**Fig. 17:**
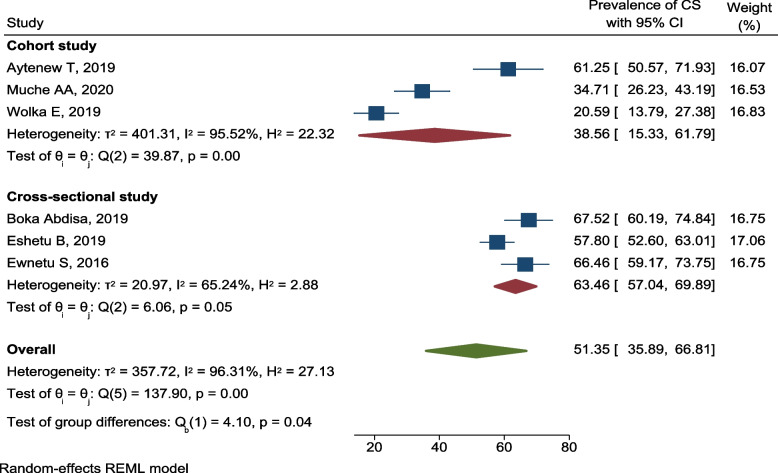
Subgroup analysis showing the prevalence of cesarean section among pregnant women with hyperglycemia based on the study design in Ethiopia, 2021

The pooled prevalence of cesarean section among pregnant women with HIP was higher and moderately variable in cross-sectional studies (63.5%; 95% CI 57.0, 69.9; *I*^2^ = 65.24). On the other hand, the cohort studies found considerably variable prevalence of that ranged from 20.6 to 61.3% with pooled prevalence of 38.6% (95% CI 15.3, 61.8; *I*^2^ = 95.52) (Fig. 17).

**Fig. 18:**
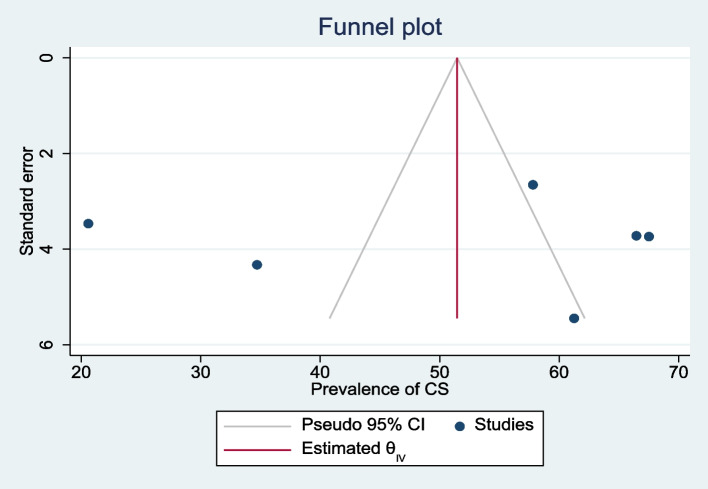
Funnel plots of the prevalence of cesarean section among pregnant women with HIP in Ethiopia, 2021

The was no publication and small-study effects which indicated by symmetry of funnel plot and Egger’s test of a *p*-value of 0.8637. The trim and fill analysis added one study and the pooled prevalence of cesarean section among HIP in Ethiopia become 57.6% (95% CI 39.7, 75.4) (Fig. 18).

## Discussion

It is crucial to assess the burden, risk factors, and pregnancy outcomes of an ever-increasing HIP in developing countries like Ethiopia. Understanding the epidemiology of HIP is imperative to improve the maternal and child health [53]. Hence, the aim of this systematic review and meta-analysis was to identify the prevalence, association factors, and feto-maternal outcomes of HIP in Ethiopia.

The prevalence of HIP among women in Ethiopia varied considerably across reports of primary studies, ranging from 0.4 [35] to 26.2% [42]. This difference might be attributed to the variation in study designs, screening method, screening criteria, testing approaches, gestational age of pregnant women during screening, study location, year of publication, and risk of bias. The availability of a standardized universal screening protocol is essential for combining the findings of various studies to produce a national, regional, or even global representative pooled prevalence of HIP [54].

The overall pooled prevalence of HIP among pregnant women in Ethiopia was 6.9% (95% CI 2.20–11.58). This finding was consistent with pooled prevalence reported in Iran (3.41%) [55], Europe (5.4%) [56], Turkey (7.7%) [57], India (8.9%) [58], SSA (9%) [16], Nigeria (11.0%) [59], and Asia (11.5%) [12]. Our result was also slightly higher than meta-analysis in Norway which reported prevalence of less than 2% [60]. However, our finding was less than similar meta-analyses in Africa (13.6%) [54], China (14.8%) [61], and Malaysia (21.5%) [62]. These discrepancies may be partly explained by differences in screening, socioeconomic factors, lifestyle, and diagnostic criteria, screening strategy, and study population. However, the main reason for this disparity may be the heterogeneity between studies.

The current meta-analysis revealed significant heterogeneity among the primary studies on HIP in Ethiopia. Similarly, several meta-analyses have consistently reported significant heterogeneity in the prevalence of GDM worldwide [12, 16, 54, 58, 59, 62–64]. Such considerable heterogeneity was reported in meta-analysis of the prevalence of DM during pregnancy in various regions, including Asia (*I*^2^ = 95%) [12], Malaysia (*I*^2^ = 95.97) [62], Africa (*I*^2^ = 96.1%) [54], SSA (*I*^2^ = 96.9%) [16], Nigeria (*I*^2^ = 99%) [59], India (*I*^2^ = 99.51%) [58], and Sub-Saharan Africa (*I*^2^ = 100) [64]. This heterogeneity may stem from a range of factors, including sociocultural, environmental, and economic factors, methodological variations (study designs, study setting, population, and patient recruitment), variations in screening methods across countries, lack of consensus on diagnostic criteria, publication year of studies (time trend), risk of bias, and differences in susceptibility to GDM among different populations. Since various diagnostic criteria were used to diagnose GDM across different areas, it is expected to observe high heterogeneity in the prevalence of DM during pregnancy in different countries.

Moreover, due to this considerable heterogeneity, which suggests the diversity of the primary studies, the pooled prevalence cannot be generalized across populations in Ethiopia. Therefore, subgroup analysis was performed to evaluate the variability in the prevalence of HIP based on different characteristics such as screening method, study design, study location, and risk of bias. However, significant heterogeneity was still observed in the subgroup analyses. Similar heterogeneity in subgroup analysis was reported in a previous meta-analysis on the prevalence of GDM in Africa [54]. Hence, it is essential to reach a consensus on a common diagnostic criterion for HIP [63].

Several factors associated with HIP have been reported in primary studies. The most identified associated factors with HIP in Ethiopia included a family history of DM, obesity, previous history of abortion, history of macrosomic baby, history of stillbirth, history of CS, parity, dietary diversity, maternal age, education status, and place of residence. However, most of these factors have been reported by only a few studies. Furthermore, there was inconsistency among the primary studies in assessing the association between these factors and DM in pregnancy. As a result, it was challenging to determine the pooled effects of each associated factor on HIP.

Several systematic reviews and meta-analyses conducted in various parts of the world [12, 16, 54, 55, 57, 59, 61, 64] have also documented similar risk factors for HIP. A meta-analysis conducted in Iran identified gestational age, history of gestational diabetes, family history of diabetes, BMI, abortions, parity, and history of macrosomia as factors associated with GDM [55]. According to a meta-analysis in Turkey, the most commonly reported risk factors were advanced maternal age, pre-pregnancy overweight, family history of DM, a history of GDM, history of giving birth to a large baby, and the number of pregnancies and births [57]. A meta-analysis in Asia also reported that a history of GDM, BMI ≥ 25, pregnancy-induced hypertension, family history of diabetes, history of stillbirth, history of abortion, age ≥ 25, multiparity ≥ 2, and a history of preterm delivery were the most important risk factors for GDM in the Asian population [12]. Similarly, the most important risk factors for GDM in SSA were advanced maternal age, history of GDM, previous stillbirth, previous macrosomia, abortion in prior pregnancies, family history of type 2 diabetes and hypertension, being older than 25 years, overweight or obese, or multipara women [16, 59, 64].

Evidence has revealed that a family history of DM is an important independent risk factor for GDM [26, 65–68].This meta-analysis also demonstrated that a family history of DM was a significant factor of HIP. This finding was consistent with meta-analyses in Asia [12], China [61], Africa [54], and SSA [16, 64]. According to these meta-analyses, pregnant women with a family history of DM were more likely to develop HIP in SSA [16, 64], Africa [54], Asia [12], and China [61]. Other meta-analyses also revealed that pregnant women with a family history of DM had more chance of developing GDM [26, 69]. This association between family history of DM and HIP suggests the genetic predisposition of HIP, which further highlights that neonates born from pregnant women with HIP will have a higher chance of developing HIP in their future. Moreover, this association might be due the common risk factors shared between GDM and DM which include obesity, family history of DM, and history of abnormal glucose tolerance [66].

Maternal obesity during pregnancy is linked to a higher risk of developing complications such as GDM and hypertension [65, 70, 71]. Obesity is one of the main risk factor for the development of HIP [66, 72, 73]. A previous meta-analysis indicated that the risk of developing GDM increases with maternal BMI [72]. However, the current meta-analysis identified no significant association between maternal obesity and HIP in Ethiopia. This finding was inconsistent with studies in Asia [12], China [61], SSA [16, 64], and Africa [54]. Meta-analyses in Asia [12] and Africa [54] reported that the chance of developing GDM was higher among pregnant women with overweight and/or obesity. This inconsistency might be due to differences in the measurement of obesity between studies. Some studies [36, 40] were assess obesity using BMI, while others [43, 50] were assess obesity using MUAC.

Gestational diabetes is associated with increased risk of a range of adverse outcomes for both fetus and mothers [66, 74–76]. There is growing evidence that GDM significantly increases the risk of adverse fatal consequences [77, 78]. Macrosomia and its associated complications are the most frequent and serious types of morbidity for infants associated with GDM [2, 78]. Macrosomia has been reported to occur in 15–45% of newborns from mothers with HIP (in comparison to 12% of newborns of normal mothers) [79]. Our meta-analysis found that 27.25% (95% CI 9.38%, 45.11%) of newborns from pregnant women with HIP in Ethiopia were macrosomic, ranging from 9.2 [35] to 78.4% [39]. This significantly high proportion of macrosomia was strikingly similar to reported in previous studies [16, 74–76, 78, 80–82]. A five-year cohort study in Iran has shown that 39.5% of diabetes subjects delivered macrosomia neonates [82]. This high proportion of macrosomia can be explained by maternal hyperglycemia leading to the passage of higher levels of blood glucose (through the placenta) into fetal circulation, which causes fetal hyperglycemia that prematurely stimulate fetal insulin secretion [83]. From the second trimester onwards, the fetal pancreas responds to hyperglycemia by secreting insulin, resulting in hyperinsulinemia. This combination of hyperinsulinemia and hyperglycemia leads to an increase in the fat and protein stores of the fetus as well as an accelerated fetal growth and fat deposition, resulting in macrosomia [79, 84].

On the other hand, a significant proportion (16.1%) of neonates from mothers with HIP in Ethiopia was born with low birth weight. This low birth weight ranged from 5.7 to 33.1%. A previous study also indicated that GDM during pregnancy was significantly associated with LBW associated with LBW [80].

Another common fetal complication identified by this study was ICU admission. Almost half (46.2%) of neonates from mothers with HIP in Ethiopia were admitted to ICU. This ICU admission was ranged from 14.9% in eastern Ethiopia to 65.3% in central Ethiopia. GDM is associated with a significantly increased risk of NICU admission [74]. Similar findings and association have been reported by previous studies [85, 86]. This higher proportion of ICU admission might be associated with higher respiratory distress, macrosomia, birth trauma, hypoglycemia, and prematurity [77, 84, 85]. Mainly, respiratory distress was the most common ICU admission diagnosis among newborns from HIP women [77, 85].

Hyperglycemia in pregnancy is associated with a significantly higher risk of preterm labor [78]. The pooled prevalence of preterm birth among mothers with HIP was 16.9%. Previous reports also demonstrated this higher risk of prematurity among women with GDM [75, 78, 83, 87]. This high risk of prematurity might be associated with early induction of labor before 39 weeks of gestation and/or premature rupture of membranes [79].

Although the reason is still unclear, infants from women with HIP have a greater risk of developing one of the most common and serious morbidity, respiratory distress syndrome [74, 75, 78]. The current meta-analysis indicated that 10.1% of newborns from mothers with HIP developed RDS in Ethiopia. Study in the USA demonstrated that 1.5–4% of newborns from mothers with GDM developed RDS [78]. Similarly, previous studies also shown that infants from women with GDM had higher risk of developing RDS [74, 75, 87]. This might be associated with the effects of hyperglycemia delays fetal lung maturity. Moreover, higher levels of glucose in utero may result in surfactant production, weak stabilization of alveoli, and responsible for development of RDS [88–90].

Women with HIP in Ethiopia were at highest risk of developing HDP, operative delivery, and PROM. Gestational diabetes and HDPs are common complications among pregnant women worldwide because they share metabolic and cardiovascular risk factors [91, 92]. The prevalence of HDP among pregnant women with HIP in Ethiopia was 28.0%. A systematic review and meta-analysis in SSA [16], South Asia [76], and Turkey [57] revealed that GDM was associated with an increased risk of pregnancy-induced hypertension among pregnant mothers. It has been reported that women with HIP are at greater risk for pre-eclampsia [2, 87, 93, 94]. Even mild HIP is associated with a significantly higher risk of HDP [95]. The development of HDP among pregnant women with HIP might be associated with multifactorial etiology, mainly insulin resistance and vascular endothelial dysfunction [91, 92, 95, 96]. These two diseases share a common pathophysiology and are characterized by systemic endothelial dysfunction [91]. This dysfunction of the vascular endothelium with dysregulation of angiogenesis plays a central role in the pathophysiology of these diseases [97]. Under normal physiological conditions, insulin activates the production of nitric oxide (NO) in endothelial cells. In insulin resistance situations, the actions of insulin in the cardiovascular system are reduced, resulting in decreased production of NO and its vasodilating action, favoring high blood pressure [96]. Moreover, insulin resistance can lead to hyperinsulinemia and beta-cell dysfunction. Prolonged hyperglycemia, insulin resistance, and dyslipidemia also affect endothelial function, leading to atherosclerosis, vascular thickening and stiffness, vasoconstriction, and its related complications [92].

The finding of the current meta-analysis revealed that half (51.4%) of pregnant women with HIP gave birth through operative delivery in Ethiopia [35, 37–39, 45, 49]. This higher proportion or risk of operative delivery among pregnant women with GDM was also has been reported from other previous studies [57, 74–76, 87, 98]. This higher rate of caesarean delivery might be associated with high incidence of macrosomia among mothers with HIP and fear of birth trauma [79, 82, 84].

## Strength and limitation

This review has certain strengths and limitations. This review included primary studies from all parts of the country. It is the comprehensive systematic review and meta-analysis on the burden, risk factors, and feto-maternal outcomes of HIP in Ethiopia. Hence, it can provide a clear picture of the epidemiology of HIP in Ethiopia. Extensive subgroup analysis using geographical distribution, screening methods, risk of bias, and study design was performed to address the heterogeneity between studies. Furthermore, risk of bias assessment and quality assessment checklists were applied to assess the risk of bias and to exclude studies with low quality.

However, our meta-analysis has limitations. First, the result of this meta-analysis had substantial heterogeneity. The screening approach and diagnostic criteria of HIP are continuously changed over time. These primary studies have been conducted in different times and combining their results can cause high heterogeneity. Second, except one community-based study, all the included studies were institution-based. Due to this, the results may reflect only those pregnant women who attended ANC follow-up. Third, including studies published only in English also has its own impact on the generalizability of this review. Even if majority of researchers published their research in English, still there is a probability of missing few articles published in other languages. Finally, although most of the articles included in this review assessed the demographic characteristics, medical factors, and obstetric factors, there were limited studies which presented the association of other variables like residence, dietary diversity, substance abuse, and physical activity issues with GDM. Due to this limitation of studies, it was difficult to determine the association between HIP and some key factors like dietary diversity, previous history of GDM, obesity, history of macrosomic baby, and history of stillbirth. Thus, those considerations must be considered in using of the results of this meta-analysis.

## Conclusion and recommendation

The heterogeneity among selected studies was significantly high which could not easily be explained by selected study characteristics and could affect the reliability and validity of the findings. Despite this heterogeneity between studies, this meta-analysis indicated that 7 out of 100 pregnant women in Ethiopia had HIP. It was also observed that there were few studies on risk factors for HIP in Ethiopia that make difficult to determine the main factors that can lead to development of HIP in Ethiopia. This meta-analysis also showed that family history of DM was a strong predictor of HIP. Macrosomia, preterm birth, stillbirths, admission to ICU, low birth weight, and respiratory distress were the most common adverse fetal outcomes whereas hypertensive disorders of pregnancy, operative delivery, and premature rapture of membrane were the main adverse maternal outcomes of pregnant women with HIP in Ethiopia.

Our study also provides an insight into the need of national guideline to direct all healthcare providers in a uniform screening of pregnant women against HIP. The healthcare professionals should educate pregnant women about risks and consequences of HIP and should take all preventive measures to reduce short- and long-term maternal and child complications related to HIP in Ethiopia. More comprehensive and representative epidemiological studies are clearly required to identify the burden, the main risk factors, and adverse pregnancy outcomes of HIP using standardized uniform screening criteria for better understanding of the disease in the in Ethiopia.

## Data Availability

The datasets analyzed during the current study are available from the corresponding author on reasonable request.

## Abbreviations

ANC: Antenatal care
APH: Antepartum hemorrhage
CI: Confidence interval
CS: Cesarean section
DM: Diabetic mellitus
GDM: Gestational diabetes mellitus
HDP: Hypertensive disorders of pregnancy
HIP: Hyperglycemia in pregnancy
ICU: Intensive care unit
IUFD: Intrauterine fetal death
OGTT: Oral glucose tolerance test
OR: Odds ratio
PPH: Postpartum hemorrhage
PROM: Premature rapture of membrane
WHO: World Health Organization
